# Computational Large Field-of-View RGB-D Integral Imaging System

**DOI:** 10.3390/s21217407

**Published:** 2021-11-08

**Authors:** Geunho Jung, Yong-Yuk Won, Sang Min Yoon

**Affiliations:** 1 HCI Lab, College of Computer Science, Kookmin Univesity, 77 Jeongneung-ro, Souel 02707, Korea; ehwk9200@kookmin.ac.kr; 2 Electronics Engineering Department, Myongji University, 116 Myongji-ro, Cheoin-gu, Yongin-si 17058, Korea; bluejerry@mju.ac.kr

**Keywords:** light field imaging, monocular depth map estimation, computational integral imaging

## Abstract

The integral imaging system has received considerable research attention because it can be applied to real-time three-dimensional image displays with a continuous view angle without supplementary devices. Most previous approaches place a physical micro-lens array in front of the image, where each lens looks different depending on the viewing angle. A computational integral imaging system with a virtual micro-lens arrays has been proposed in order to provide flexibility for users to change micro-lens arrays and focal length while reducing distortions due to physical mismatches with the lens arrays. However, computational integral imaging methods only represent part of the whole image because the size of virtual lens arrays is much smaller than the given large-scale images when dealing with large-scale images. As a result, the previous approaches produce sub-aperture images with a small field of view and need additional devices for depth information to apply to integral imaging pickup systems. In this paper, we present a single image-based computational RGB-D integral imaging pickup system for a large field of view in real time. The proposed system comprises three steps: deep learning-based automatic depth map estimation from an RGB input image without the help of an additional device, a hierarchical integral imaging system for a large field of view in real time, and post-processing for optimized visualization of the failed pickup area using an inpainting method. Quantitative and qualitative experimental results verify the proposed approach’s robustness.

## 1. Introduction

**Figure 1:**
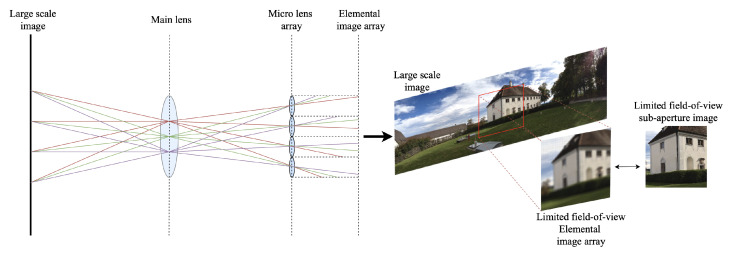
Typical integral imaging pickup system that has limited field of view.

The integral imaging system has played an important role in the field of three-dimensional (3D) displays, creating a light field using two-dimensional (2D) micro-lens arrays. Integral imaging that incorporates autostereoscopic and multiscopic imaging provides 3D images through a micro-lens array because they do not require users to wear glasses and offer more viewing flexibility for 3D broadcasting, real-time motion imaging, virtual/augmented reality, etc. Lippmann [1] introduced the concept of integral imaging to provide a 3D display using different perspectives according to the viewing direction. Traditional integral imaging systems [2] have been based on pickup and display procedures. The pickup stage records a ray emitted from the object onto an image sensor through each micro-lens center. Thus, the system generates many packed distinct elemental images, which are then viewed through an array of convex lenses or pinholes. The display stage passes the ray from the elemental image array through each micro-lens center, producing a 3D autostereoscopic image from an elemental image array. However, traditional integral imaging methods using physical micro-lens arrays have two limitations. First, it is difficult to set the micro-lens arrays to the exact positions to avoid the interference of rays between different micro-lenses. Furthermore, more complex and larger micro-lens arrays are needed to generate a desirable elemental image array from a large-scale object without distortion. To tackle these limitations, some approaches used pixel mapping [3,4,5,6,7]. Li et al. [7] proposed a simplified integral imaging pickup method using a depth camera. The computational integral imaging method takes a pair of RGB images and a depth image obtained from the KINECT as inputs, and calculates pixel mapping from the object image plane to the elemental image plane. Recent computational integral imaging pickup systems also attempt to overcome the above limitations without prior information or auxiliary devices, but they struggle to obtain a large-field-of-view (FOV) sub-aperture image without distortion [3,4,5,6,7]. As shown in Figure 1, current integral imaging systems using physical or virtual micro-lens arrays still have problems in representing large FOV images, from panoramic images to fisheye images. Since integral imaging systems are affected by the micro-lens array size, number of micro-lenses in the lens array, or input image resolution, resultant sub-aperture images have low resolution and small FOV when applying the system to large-scale images, such as high-resolution or panorama images, where a sub-aperture image can be obtained by transposing the pixels in each elemental image (light field parametrization [8]).

The second limitation of the existing computational integral imaging system is the processing time. Most proposed methods have accelerated the processing time of generating an elemental image array with the help of a GPU, but they are still restricted to real-time applications for small object images.

**Figure 2:**
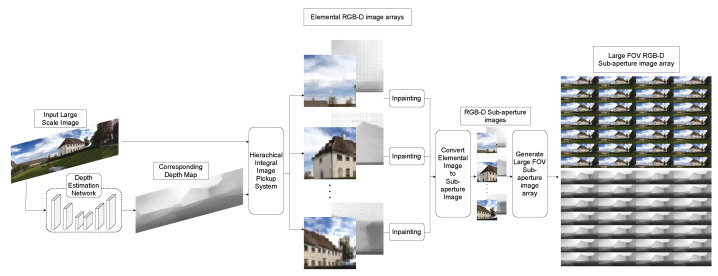
Proposed large-field-of-view RGB-D integral imaging pickup system comprising automatic depth map estimation and hierarchical integral imaging pickup system.

To overcome the major limitations of the previous physical and computational integral imaging methodology, as well as to provide convenience for users to generate large elemental image arrays without supplementary devices, we propose a large-FOV integral imaging pickup system from a single image in real time. Specifically, we utilize the hierarchical integral imaging pickup system, where multiple virtual lens arrays work on different parts of the given large-scale image to improve the performance of the current integral imaging system and to obtain a sub-aperture RGB-D image with a large FOV. The resulting images are integrated into a large-FOV elemental image array, which generates large-FOV sub-aperture images. In particular, the computational deployment of the virtual micro-lens arrays in an overlapped manner enables us to provide seamless large-FOV integral images, as well as to minimize the failed pickup area during the procedure. Figure 2 shows the proposed large-FOV integral imaging pickup system. We first estimate the depth map using a deep neural network (DNN) embedding attention network to emphasize meaningful features while reducing redundant noise from a given input image. We also propose a hierarchical integral imaging pickup system to extend the FOV. Finally, we apply post-processing by inpainting failed pickup areas (FPAs) for better human visual perception.

The major contributions of this study can be summarized as follows:

- We present an end-to-end computational large-FOV RGB-D integral imaging pickup system from single input images using a hierarchical integral imaging pickup procedure;
- The proposed RGB and depth integral imaging pickup system computationally eliminates the distortion of elemental images and contamination;
- We present quantitative and qualitative analyses for the proposed method to be applied to various applications in real time.

The remainder of this paper is organized as follows. Section 2 explains the proposed algorithm in detail. Section 3 verifies the proposed algorithm experimentally. Section 4 summarizes and concludes the paper.

## 2. Large-Field-of-View Rgb-D Integral Imaging System

This section explains the technical details regarding extracting depth maps from a single image and designing the hierarchical integral image pickup system to provide a large-FOV elemental RGB-D image array and sub-aperture image array. The hierarchical integral RGB-D image pickup process to provide a large FOV comprises two stages: multiple computational shift lens array manipulation within a virtual main lens, and sub integral RGB-D image pickup for each virtual shift lens. We also describe how to overcome FPA using inpainting to obtain improved elemental imaging visualization, where we assume that the main lens is a virtual lens for generating sub-aperture images [9,10].

### 2.1. Multi-View Attention Module-Based Monocular Depth Map Estimation

Stereo vision captures two different scene views in a manner similar to the human vision system and has been used to estimate the depth map in the area of computer vision. Although stereo vision is becoming increasingly common, there are still barriers to accurately estimating the depth map, including barrel and tangential distortion adjustment, image rectification, and automatic feature extraction and matching procedures. The proposed approach estimates the depth map from a given single image without assumptions or supplementary devices by employing machine learning.

**Figure 3:**
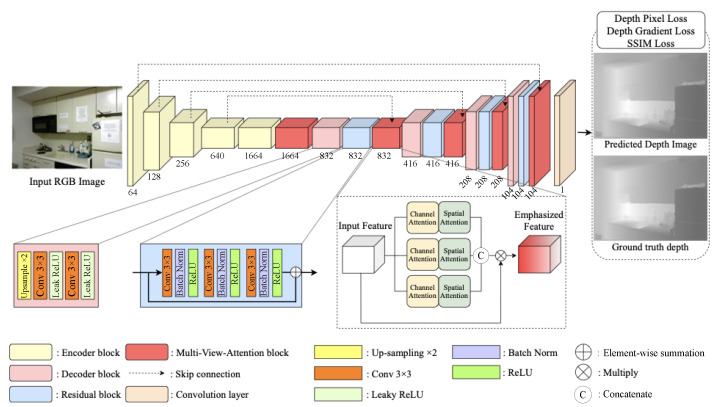
Deep neural network architecture for monocular-image based depth estimation.

We propose DNN-based depth estimation from a single image by training a huge number of RGB-D datasets, such as the NYU-V2 RGB-D dataset [11]. The dataset consists of video sequences of various indoor scenes, which are captured by Microsoft Kinect, and each RGB image is paired with its depth image. Our proposed network is trained with this dataset and the RGB images and the depth images are used as the input and the ground-truth images, respectively. The proposed DNN comprises encoder and decoder blocks with multi-view attention (MVA) modules by extending channel and spatial attention from the 3D feature map [12]. Convolution layers in the encoder extract features from input images for robust depth estimation and are subsequently down-sampled by pooling layers in the encoder phase. Therefore, we obtain the latent features to reconstruct a depth map using the DNN. The decoder passes latent features through convolution and up-sampling layers to reconstruct a depth map corresponding to the RGB image. Feature maps from the decoder block pass through the MVA block to emphasize meaningful features and reduce redundant features. Encoder and MVA blocks are connected by skip connections to update the features at the appropriate rate. Figure 3 shows that we obtain a depth map corresponding to the input RGB image by passing through various DNN blocks. Table 1 describes the details of the proposed architecture of the MVA network, whose kernel size is set to 3×3, and stride is 1.

To effectively train and optimize the proposed network, we define the loss function (Loss) to minimize differences between ground-truth and predicted depth maps by including pixel-wise, depth image gradient, and structural similarity index measure (SSIM) loss functions, i.e.,

(1) $$Loss\left(y,\hat{y}\right)=\lambda Loss_{d}\left(y,\hat{y}\right)+Loss_{g}\left(y,\hat{y}\right)+Loss_{SSIM}\left(y,\hat{y}\right),$$

where we set λ=0.1 in Loss empirically.

Pixel-wise loss $Loss_{d}$ can be obtained by averaging the difference between ground-truth and predicted depth map,

(2) $$Loss_{d}\left(y,\hat{y}\right)=\frac{1}{n}\sum_{p}^{n}|y_{p}-{\hat{y}}_{p}|.$$

We calculate ground-truth and predicted depth gradients and then define the depth gradient loss $Loss_{g}$ as the difference between the ground-truth and predicted gradient. To obtain $g_{x}$, we calculate a difference between the next pixel location (x+1,y) and the current location (x,y) of the ground-truth and predicted depth image, respectively. Then, $g_{x}$ is calculated on the difference between the x-axis ground-truth gradient and the predicted depth gradient. $g_{y}$ is also calculated in the same way with respect to the y-axis gradient, which is calculated from the difference between (x,y+1) and (x,y):

(3) $$Loss_{g}\left(y,\hat{y}\right)=\frac{1}{n}\sum_{p}^{n}|g_{x}\left(y_{p},{\hat{y}}_{p}\right)\left|+\right|g_{y}\left(y_{p},{\hat{y}}_{p}\right)|.$$

Finally, we use the SSIM loss [13] $Loss_{SSIM}$, which seeks to learn to produce a visually pleasing depth map image, by measuring the structural similarity of the depth map images, compares the local texture of the ground truth and output images, and adjusts the small spatial misalignment. $Loss_{SSIM}$ is shown as follows:

(4) $$Loss_{SSIM}\left(y,\hat{y}\right)=\frac{1-SSIM(y,\hat{y})}{2}.$$

The final loss function Loss combines $Loss_{d}$, $Loss_{g}$, and $Loss_{SSIM}$ to effectively optimize the cost over the entire training RGB-D images dataset.

The proposed DNN depth map estimation by embedding MVA blocks extracts and enhances meaningful features in each layer to prevent blurring and staircase effects around discontinuous depth areas with significantly improved performance. The system can accurately estimate depth maps even with partial occlusions, illumination changes, and background clutter. We employ hierarchical integral imaging pickup from the RGB input image and corresponding depth map.

### 2.2. Hierarchical Integral RGB-D Imaging System

**Figure 4:**
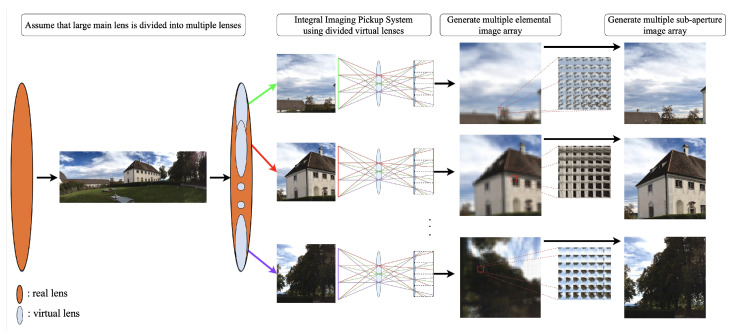
Proposed hierarchical integral imaging pickup system comprising multiple shift-lens arrays and sub-integral imaging pickup for each shift lens.

As shown in Figure 4, we place the micro-lens array hierarchically for a given large-scale image or a panorama image. The hierarchical integral RGB-D imaging system includes two stages, assuming multiple computational shift-lens array and sub-integral imaging pickup systems for each virtual shift lens. We shift the virtual lens array within the given main lens for large-scale input images to look at various input image parts.

#### 2.2.1. Multiple Shift-Lens Array Manipulation Process

The proposed approach applies a computational integral imaging pickup system to large-scale or panoramic images, generating large sub-aperture and elemental image arrays to effectively capture sub-areas from the given input image. We first divide the main lens sectioning the large-scale input image into several sub main lenses that look at various input image parts, with each shift-lens array providing input to the integral imaging pickup system. Thus, we generate the large-scale elemental image array from multiple shift-lens arrays. The divided virtual lenses can be expressed as

(5) $$M\to M_{1},M_{2},M_{3},\ldots ,M_{n}$$

and

(6) $$E\to E_{1},E_{2},E_{3},\ldots ,E_{n},$$

where *M* is the large virtual lens with corresponding large micro-lens array *E*, and each part of the whole image *I* corresponding a $E_{n}$ is expressed as $I_{n}^{part}$.

(7) $$I\to I_{1}^{part},I_{2}^{part},I_{3}^{part},\ldots ,I_{n}^{part}$$

We define the resulting elemental image arrays by adding each generated $A_{i}$ as shown below:

(8) $$I^{EIA}=\sum_{i=1}^{n}A_{i}\left(I_{i}^{part},E_{i}\right)$$

where $I^{EIA}$ is the integrated large-scale elemental image array. We implement virtual and multiple shift-lens arrays to be slightly overlapping in order to avoid missing rays emitted from the images.

#### 2.2.2. Sub-Integral Imaging Pickup Process

Each input image part is then presented to the integral imaging pickup system by the divided micro-lens arrays. Previous methods used physical micro-lens array(s), whereas we propose virtual micro-lens arrays to calculate pixel mapping from image to elemental image pixel coordinates. We extend Li’s method [7], using deep learning-based depth estimation from a single RGB image, rather than a depth camera, to obtain depth maps corresponding to input RGB images. The central depth value *d* in a valid depth range of the integral imaging system and pixel size for input image $P_{I}$ are calculated as $d=\frac{f\cdot g}{f+g}$ and $P_{I}=\frac{d}{g}\cdot P_{D}$, respectively, where *f* is the micro-lens array focal length, *g* is the distance between display and micro-lens array, and $P_{D}$ is the monitor’s pixel pitch. Thus, the valid depth range can be expressed as

(9) $$\Delta d=\frac{2\cdot d}{P_{L}}\cdot P_{I},$$

where $P_{L}$ is the micro-lens pitch and Δd represents the depth range expressible in the integral imaging system. From Equation (9), the converted depth map can be expressed as

(10) $$L_{\left(i,j\right)}=\frac{d\cdot (max(Z)+min(Z))}{Z_{\left(i,j\right)}\times 2},$$

where *Z* is a depth map, and $Z_{\left(i,j\right)}$ is the depth at pixel (*i*, *j*).

**Figure 5:**
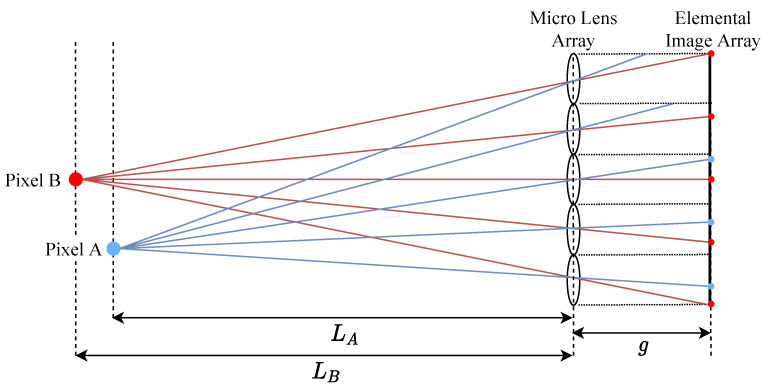
Pixel mappings from image to elemental image array coordinates.

Figure 5 shows a ray emitted from a pixel passing through the micro-lens center to its location in each elemental image. Elemental image pixel coordinates (u,v) are defined as

(11) $$u=P_{L}\cdot i_{L}-\left(i\cdot P_{I}-P_{L}\cdot i_{L}\right)\cdot \frac{g}{L_{\left(i,j\right)}}$$

and

(12) $$v=P_{L}\cdot j_{L}-\left(j\cdot P_{I}-P_{L}\cdot j_{L}\right)\cdot \frac{g}{L_{\left(i,j\right)}}.$$

Each micro-lens array generates elemental image arrays using this method [7], without requiring a physical micro-lens array.

### 2.3. Postprocessing to Eliminate Failed Pickup Areas

**Figure 6:**
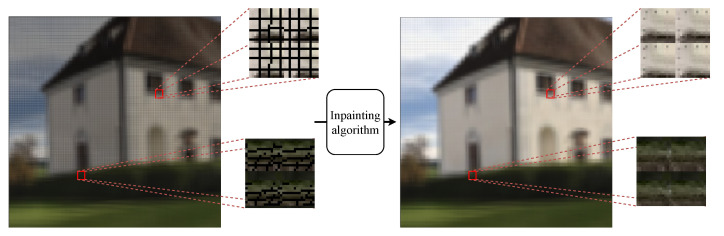
Elemental images with failed pickup area and resultant images from inpainting.

Computational hierarchical integral imaging systems effectively reduce traditional optical pickup problems. However, FPA still affects elemental images’ and sub-aperture images’ visualization, i.e., empty areas between neighboring object points from the micro-lens array. Figure 6 represents FPA as black lines and/or regions. We apply inpainting to replace FPA with neighboring pixels for human visual perception using the Navier–Stokes equation from fluid dynamics to calculate partial differential equations to extract edges from known to FPA regions. This preserves continuous isophotes while matching gradient vectors at inpainting region boundaries [14], effectively providing color information for FPA regions while reducing the minimum variance across the area. Figure 6 shows an example FPA and inpainted result. The pseudo-code of the large-FOV integral imaging is represented in Algorithm 1. Section 3 details experiments to investigate the proposed integral imaging method’s efficiency.
**Algorithm 1** Proposed system**Input:** I: Inputted RGB image         Φ: Monocular depth estimation network         M: Virtual main lens         E: Virtual micro-lens array         **A**: Sub integral imaging pickup process function         **S**: Convert function from elemental image array to sub-aperture image array**Output: **$I^{SA}$, $D^{SA}$, Large FOV sub-aperture image array about image *I* and depth *D*1: $I^{EIA}\leftarrow \emptyset$**;**$D^{EIA}\leftarrow \emptyset$2: D←Φ(I)                                                                            ▹*D* is a predicted depth3: $I\to \{I_{1}^{part},I_{2}^{part},\ldots ,I_{n}^{part}\}$                            ▹*I* is divided into set of n $I_{n}^{part}$4: $D\to \{D_{1}^{part},D_{2}^{part},\ldots ,D_{n}^{part}\}$                           ▹*D* is divided into set of n $D_{n}^{part}$5: $M\to \{M_{1},M_{2},\ldots ,M_{n}\}$                                         ▹*M* is divided into set of n $M_{n}$6: $E\;\to \{E_{1},E_{2},\ldots ,E_{n}\}$                                               ▹*E* is divided into set of n $E_{n}$5: **for** i←0**to***n*   **do**6:    **if** $I_{i}^{part}\in I$ **and** $D_{i}^{part}\in D$ **then**7:       $I_{i}^{EIA}\leftarrow A_{i}\left(I_{i}^{part},E_{i}\right)$8:       $I^{EIA}\leftarrow I^{EIA}\cup I_{i}^{EIA}$9:       $D_{i}^{EIA}\leftarrow A_{i}\left(D_{i}^{part},E_{i}\right)$10:     $D^{EIA}\cup D_{i}^{EIA}$11: **end for**12: $I^{SA}\leftarrow$ **S** $\left(I^{EIA}\right)$13: $D^{SA}\leftarrow$ **S** $\left(D^{EIA}\right)$14: **return $I^{SA},D^{SA}$**

## 3. Experiments

This section presents quantitative and qualitative experimental results to verify the robustness of the proposed RGB-D integral imaging system.

### 3.1. Implementation Details

The proposed system was implemented in Python PyCuda on a NVIDIA GeForce GTX 1070 GPU using the TensorFlow2 framework to provide a real-time response. For monocular depth estimation using MVA blocks to emphasize the important features as well as reduce the redundant features from the input RGB image, we used DenseNet-169 [15], pre-trained on ImageNet [16], as the encoder for our monocular depth estimation network to extract dense features. Batch size was set to 4 and the network was trained for 20 epochs using the ADAM optimizer with learning rate = 0.0001. Our network was trained with the 50 K NYU-V2 RGBD images dataset.

For fair performance evaluation in the large-FOV integral imaging pickup system, we set virtual micro-lens array focal length = 10 mm; gap between micro-lens array and display = 11 mm; LCD monitor pixel pitch = 0.1245 mm; micro-lens size = 1.8675 mm; input RGB image using real lens has 2000×700 resolution; and number of lenses in each virtual micro-lens array = 62,500 (250×250). Thus, each elemental image array from the multiple shift-lens array generates 3750×3750 resolution. The proposed system integrates each elemental image array, resulting in a 15,750×5700 final elemental image array.

### 3.2. Qualitative and Qualitative Analysis

We first verified our proposed MVA-based monocular depth estimation network by quantitatively comparing various depth estimation algorithms using error matrices, including average relative error (REL), root mean squared error (RMS), and average ($\log_{10}$) error. Table 2 shows that the proposed network produces remarkable depth maps. The proposed depth map prediction network embedding MVA blocks effectively emphasize the depth feature as well as remove redundant features to estimate the depth map from the single image. In particular, the proposed depth map estimation network provides clear differences around object boundaries. Feature enhancement in the DNN considerably improves the depth map accuracy, as shown in Table 2. Since the depth map is closely related to the distortion of the integral imaging system, the proposed MVA-based depth map can contribute to improving the accuracy of the conventional physical and computational integral imaging system.

**Figure 7:**
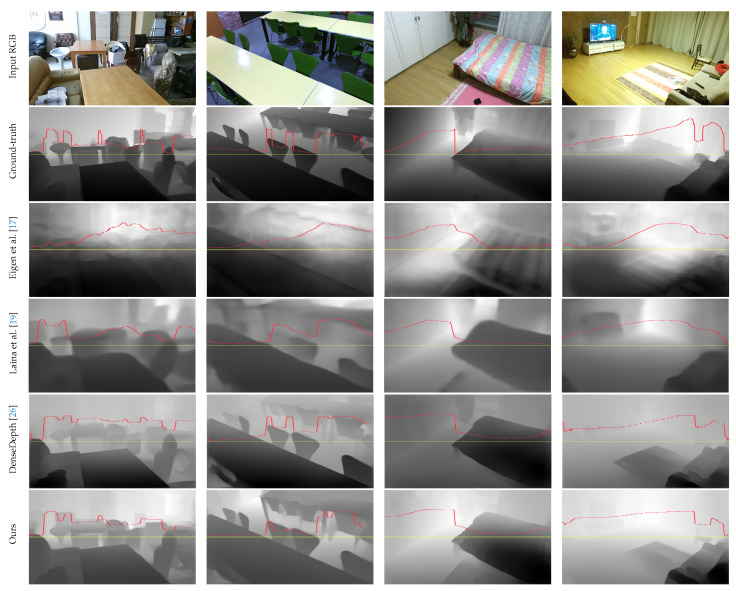
Example images of monocular depth estimation using deep neural network. For a fair and easy comparison, depth intensity values were extracted from each image along the same positioned scanline.

Figure 7 shows the qualitative comparison of the monocular depth estimation prediction results using the DIML/CVL RGB-D dataset (https://dimlrgbd.github.io/downloads/technical_report.pdf, accessed on 3 August 2021) [27,28,29], which includes indoor and outdoor RGB-D images. It shows input RGB images (1344×756) (first row of Figure 7), corresponding ground-truth depth maps (second row of Figure 7), and predicted depths from the proposed network, respectively. Predicted depth maps preserved object boundaries, and depth information was reconstructed well compared with ground-truth depth maps. For a fair and easy comparison between our approach and previous approaches, the intensity values of the depth map were extracted from each depth map image along the same positioned yellow scanline in Figure 7. Within the depth layer, all methodologies maintained the depth information satisfactorily, but there were difference in separating the depth layers clearly. In particular, the depth map change of the scanline of the depth map, such as Eigen et al. [17] and Laina et al. [19], still showed oversmoothing between depth layers. On the other hand, our proposed depth map retains the prominent edges and shading without unwanted noisy information because the MVA blocks and loss functions optimized the network to generate well-matched depth maps.

**Figure 8:**
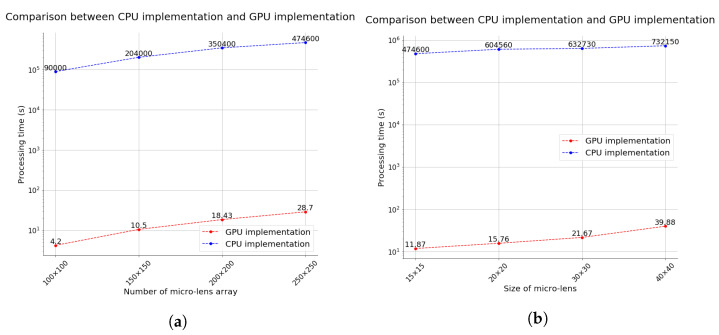
Comparison between CPU and GPU implementation for various micro-lens configurations. (**a**) Comparison between CPU and GPU implementation with respect to the number of lenses in micro-lens arrays. (**b**) Comparison between CPU implementation and GPU implementation with respect to lens size in the micro-lens arrays.

Previous integral imaging pickup systems on the CPU are difficult to apply widely since they require considerable processing time to generate each elemental image array of the large-FOV image. Therefore, we implemented parallel processing through the GPU to provide the real-time RGB-D integral imaging visualization of the large-FOV images. One pixel passes through all micro-lenses; hence, the system processes this simultaneously, enormously reducing the processing time. Figure 8 compares CPU and GPU implementations for various micro-lens configurations. Processing time increases gradually as the number of micro-lenses increases (Figure 8a) and processing time also increases as the micro-lens size increases (Figure 8b). The results shown in Figure 8 highlight the trade-off between the number of micro-lenses and processing time, and between the micro-lens size and processing time. A larger micro-lens size means that we can observe more image parts, but the overall resolution is reduced. We can effectively control the processing time even though we rapidly increase the number of micro-lenses or extend the resolution of each micro-lens by implementing the proposed RGB-D integral imaging system on the GPU.

**Figure 9:**
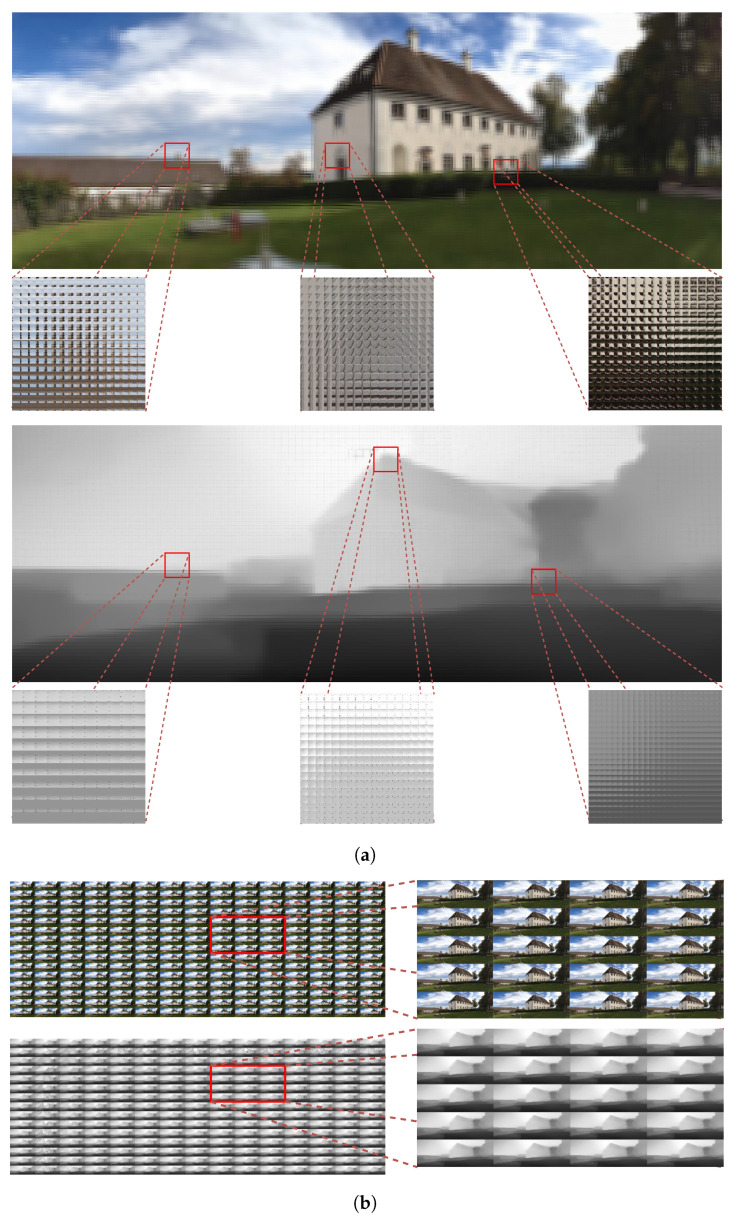
Large-field-of-view RGB-D elemental and sub-aperture image arrays example image. (**a**) RGB-D elemental image array with large field of view from the proposed method. (**b**) Sub-aperture RGB-D image array with large field of view from the proposed method.

Figure 9 presents an example of the resulting RGB-D elemental image array of the proposed method and its sub-aperture RGB-D image array to effectively visualize the proposed approach. Each shift-lens array generates an elemental image array that is slightly overlapped in order to avoid missing rays emitted from the images. These elemental image arrays are subsequently integrated by the proposed implementation, producing a 15,750 × 5700 final elemental image array resolution, as shown in Figure 9a. Thus, we can generate large-FOV elemental image arrays using virtual micro-lens arrays and high computing power, and FPAs in the elemental images are successfully removed by inpainting. Each elemental image generates properly; hence, the observed image is not reversed, as shown in Figure 9a. Pixels at the same position in each elemental image array were rearranged in the sub-aperture image array location to generate sub-aperture image arrays.

As the number of sub-aperture images is dependent on the size of the micro-lens, the resolution of the sub-aperture image is determined by the number of micro-lenses in the lens array. Figure 9b shows the number of sub-aperture images = 225 with 1050×380 pixel resolution, and Figure 9b also confirms that the proposed generates high-quality sub-aperture images. Thus, the proposed approach can overcome physical micro-lens array or depth camera limitations using advanced computing hardware, i.e., parallel processing on GPUs, and DNN learning. The quantitative and qualitative evaluation results prove that our proposed RGB-D integral imaging system can be used in real time, due in part to compelling applications in virtual and augmented reality from the given RGB input image.

## 4. Conclusions

This paper proposes an end-to-end monocular image-based large-FOV RGB-D integral image system in real time. The hierarchical integral imaging system was designed for a large FOV and included multiple shift-lens arrays and sub-integral image pickup processes. In contrast with previous physical micro-lens-based integral imaging systems, we used a computational monocular image-based integral system to minimize distortions due to physical lens array mismatches, without requiring supplementary devices.

Experimental results verified that the proposed computational integral imaging system performed favorably for large-FOV images, including panoramic images, and could be operated in real time using GPU parallel processing. The proposed large-FOV integral imaging system can be applied to various areas of computer vision and computer graphics, such as synthetic aperture imaging, segmentation and matting, object detection and classification, stitching and deblurring of images, and handling reflective and transparent objects. In particular, the proposed large-FOV integral imaging system combined with deep learning may not only improve the accuracy of the existing methodologies but also expand the scope of applications.

## Figures and Tables

**Table 1:** Detailed architecture of our proposed multi-view deep learning-based depth map estimation from a single image.

Module	Block Type	Output Dimension
**Input Image**	**-**	H×W×3
Encoder	Block 1	120×160×64
	Block 2	60×80×128
	Block 3	30×40×256
	Block 4	15×20×640
	Block 5	15×20×1664
Decoder	MVA Block	15×20×1664
	Block 1	30×40×832
	Residual Block 1	30×40×832
	MVA Block 1	30×40×832
	Block 2	60×80×416
	Residual Block 2	60×80×416
	MVA Block 2	60×80×416
	Block 3	120×160×208
	Residual Block 3	120×160×208
	MVA Block 3	120×160×208
	Block 4	240×320×104
	Residual Block 4	240×320×104
	MVA Block 4	240×320×104
	Conv 3×3	240×320×1

**Table 2:** Depth map estimation using various error measurement metrics for the NYU V2 dataset. The top two methods are highlighted in red and blue, respectively.

Method	$\delta_{1}$	$\delta_{2}$	$\delta_{3}$	REL	RMSE	$\log_{10}$
	Higher Is Better			Lower Is Better		
Eigen et al. [17]	0.769	0.950	0.988	0.158	0.641	−
Liu et al. [18]	0.614	0.883	0.971	0.230	0.824	0.095
Laina et al. [19]	0.811	0.953	0.988	0.127	0.573	0.055
Cao et al. [20]	0.646	0.892	0.968	0.232	0.819	0.091
Li et al. [21]	0.788	0.958	0.991	0.143	0.635	0.063
Xu et al. [22]	0.811	0.954	0.987	0.121	0.586	0.052
Lee et al. [23]	0.815	0.963	0.991	0.139	0.572	−
DORN [24]	0.828	0.965	0.992	0.115	0.509	0.051
Chen et al. [25]	0.826	0.964	0.990	0.138	0.496	−
DenseDepth [26]	0.846	0.974	0.994	0.123	0.465	0.053
Proposed method	0.853	0.970	0.992	0.125	0.458	0.053

## Data Availability

Not applicable.
